# Cardiovascular Biomarkers in Chronic Kidney Disease: State of Current Research and Clinical Applicability

**DOI:** 10.1155/2015/586569

**Published:** 2015-04-05

**Authors:** Luis D'Marco, Antonio Bellasi, Paolo Raggi

**Affiliations:** ^1^Unidad Avanzada de Investigación y Diagnostico Ecográfico y Renal, Clínica Puerto Ordaz, Puerto Ordaz, Venezuela; ^2^U.O.C. di Nefrologia e Dialisi, Ospedale Sant'Anna, Azienda Ospedaliera Sant'Anna, Como, Italy; ^3^Department of Health Sciences, University of Milan, Milan, Italy; ^4^Mazankowski Alberta Heart Institute, University of Alberta, Edmonton, AB, Canada; ^5^Department of Radiology, Emory University, Atlanta, GA, USA

## Abstract

The high incidence of cardiovascular events in chronic kidney disease (CKD) warrants an accurate evaluation of risk aimed at reducing the burden of disease and its consequences. The use of biomarkers to identify patients at high risk has been in use in the general population for several decades and has received mixed reactions in the medical community. Some practitioners have become staunch supporters and users while others doubt the utility of biomarkers and rarely measure them. In CKD patients numerous markers similar to those used in the general population and others more specific to the uremic population have emerged; however their utility for routine clinical application remains to be fully elucidated. The reproducibility and standardization of the serum assays are serious limitations to the broad implementation of these tests. The lack of focused research and validation in randomized trials rather than *ad hoc* measurement of multiple serum markers in observational studies is also cause for concern related to the clinical applicability of these markers. We review the current literature on biomarkers that may have a relevant role in field of nephrology.

## 1. Introduction

The very high incidence of cardiovascular disease (CVD) events and premature mortality in patients with chronic kidney disease (CKD) [1], with a sharp increase in risk as glomerular filtration rate (GFR) declines below 60 mL/min/1.72 m^2^ [2], offers a rationale for better risk stratification in this population. Several traditional risk factors and factors more closely related to loss of renal function (anemia, oxidative stress, inflammation, and bone mineral disorders) contribute to the high incidence of cardiovascular complications seen in patients with CKD. Whether biomarkers help improve the identification of patients at risk of cardiovascular events has been at the core of extensive research in the general population and in patients with CKD [3]. This approach predicates that an accurate assessment of cardiovascular risk at an early stage would facilitate more aggressive and focused treatment of those in greater need of preventive measures with the goal to reduce event rates. In this review, we focus on established and emerging laboratory biomarkers for the assessment of risk in CKD and compare them to their use in the general population.

## 2. Natriuretic Peptides

The natriuretic peptides are a family of hormones that play a major role in sodium and body volume homeostasis; specifically they control natriuresis, vasodilatation, and diuresis [4]. Three major natriuretic peptides have been identified: atrial natriuretic peptide (ANP), B-type natriuretic peptide (BNP), and C-type natriuretic peptide (CNP). They all share a common 17-amino-acid ring structure and have actions that are targeted at protecting the cardiovascular system from the effects of volume overload [5]. While ANP is preferentially produced in and secreted by the atria, BNP is produced by the ventricular myocardium in response to ventricular stretching and wall stress [6]. CNP, derived primarily from endothelial cells, is also synthesized by the myocardium. Upon ventricular myocyte stretch, pre-proBNP is enzymatically cleaved to proBNP and released as active BNP hormone (amino acids 79–108) or an inactive fragment, NT-proBNP (amino acids 1–76, released in a 1 : 1 ratio).

Natriuretic peptides, in particular BNP and NT-ProBNP, have been investigated as biomarkers in several conditions and an increase in their serum levels has been associated with degree of left ventricular dysfunction, severity of congestive heart failure symptoms, and ultimately a poor prognosis in community-based and general population studies [7–11]. Furthermore, NT-proBNP is a good marker for prediction of first cardiovascular events in the population, as well as the risk of stroke in patients with atrial fibrillation [12, 13]. NT-proBNP has a longer half-life and thus its levels may be more stable (less affected by acute stress) than BNP. Both BNP and NT-proBNP are eliminated only to a small degree during a hemodialysis session [14], and NT-proBNP appears to accumulate to a larger degree during dialysis [15, 16]. Several studies have confirmed that both BNP and NT-proBNP are useful markers of cardiovascular risk in CKD patients. In general, they have been shown to correlate with the severity of heart failure and left ventricular dysfunction and to be useful in guiding the management of heart failure in CKD. Plasma BNP concentrations increase progressively with decreasing renal function, and this relationship remains present when patients are subdivided into systolic and diastolic heart failure (*P* < 0.01) [17]. In a prospective evaluation of 213 CKD nondialysis patients, Vickery and coworkers demonstrated that NT-proBNP (≥89 pmol/L, HR 2.5, *P* < 0.05) and high-sensitivity C-reactive protein (hsCRP ≥ 4.7 mg/L, HR 1.9, *P* < 0.05) were independently associated with increased all-cause mortality [3]. Similar findings were reported in dialysis patients [18]. Additionally, NT-proBNP, but not BNP, was an independent predictor of death in a heart failure population with eGFRs < 60 mL/min/1.73 m^2^ [19].

BNP was prospectively studied as a biomarker of cardiovascular events in CKD patients in a community-based study in Japan [20]. The investigators collected baseline plasma BNP, serum creatinine, and urinary protein levels from 9625 patients. The risk of cardiovascular events was significantly higher in participants with the highest BNP serum levels. In a study of 134 hemodialysis patients Sommerer et al. found that plasma levels of NT-proBNP were elevated in 100% and cardiac troponin T (cTNT) levels in approximately 40% of asymptomatic patients [21]. Both increased NT-proBNP and cTNT were strongly associated with an adverse cardiovascular outcome (overall there were 23 deaths due to myocardial infarction and sudden cardiac death). In a prospective cohort study of 230 patients undergoing chronic peritoneal dialysis, Wang et al. reported that NT-pro-BNP was an independent risk marker of congestive heart failure, death, or a combined end-point including death and other adverse cardiovascular outcomes [22] (odds ratios varied between 4.25 and 9.1 for the fourth compared to the 1st quartile of NT-pro-BNP). The authors concluded that in chronic peritoneal dialysis patients NT-pro-BNP adds prognostic information beyond that contributed by left ventricular hypertrophy, systolic dysfunction, and other conventional risk factors. Finally, NT-pro-BNP is a predictor of mortality in CKD patients independent of volume overload and dialysis modality [23, 24].

## 3. Cardiac Troponin T

Troponin (TN) T, I, and C are components of the contractile apparatus of striated muscle. Specific forms of troponins T and I are present in the heart muscle, namely, cTNT and cTNI. cTNI is exclusively expressed in cardiomyocytes and is released into the circulation after myocardial cell damage. Increased levels of troponins are detectable 3–12 h after myocardial injury, with the concentration being in direct proportion to the extent of injury [5]. The serum levels return to the normal range after 5–14 days from injury, which is 4 times longer than for the creatine kinase myocardial band isoenzyme (CK-MB) fraction, probably because of sustained release of structurally-bound protein from disintegrating myofibrils [25].

cTNT is cardiac specific and is not present in the serum following nonmyocardial muscle damage. The levels of cTNT are commonly increased among patients with renal dysfunction in the absence of acute myocardial infarction [26]. Although the exact mechanism is unknown, several studies suggested that increased levels of troponins are associated with increased risk of coronary artery disease (CAD) and death in predialysis populations [27]. Several explanations, although controversial, have been proposed to understand cTNT elevation in uremic patients. Among them are increased left ventricular wall stress, acute or chronic volume overload, silent plaque rupture in the presence of diffuse coronary atherosclerosis, and apoptosis of cardiomyocytes [28, 29]. The prevalence of elevated cTNT plasma levels prior to hemodialysis sessions varies between 20% and 53%. A serum level of cTNT > 0.01 microg/L is relatively common (28,2%) in patients with CKD stage 3-4 [26]. A cohort of 847 dialysis patients was followed in a multicenter study in the Netherlands between 1997 and 2001. After an average follow-up of two years patients with baseline cTNT levels between 0.05 and 0.10 microg/L showed a hazard ratio for all-cause death of 2.2 (95% CI, 1.7 to 2.8) compared with patients with levels below 0.05 microg/L [30]. For those with levels greater than 0.10 microg/L (11%), the hazard ratio rose to 3.3 (95% CI, 2.5 to 4.5). In this investigation there was no significant difference in risk of events between patients receiving hemodialysis or peritoneal dialysis and between patients with high and low residual renal function. Cardiac TNT was used as a marker to identify occult obstructive coronary artery disease (CAD) in 142 patients at the start of their renal replacement therapy [31]. Of 60 asymptomatic patients at the time of evaluation, 35 (43.8%) had obstructive CAD and 27 had multivessel CAD as assessed by invasive coronary angiography. On stepwise logistic regression analyses cTNT was an independent predictor of asymptomatic multivessel CAD (sensitivity and specificity of cTNT to predict multivessel CAD: 92.6% and 63.6%, resp.). Several publications have further supported the value of cTNT as a predictor of unfavorable CAD events in CKD populations [32–34]. cTNT and cTNI were found to be equivalent in differentiating short-term prognosis in patients with CKD suffering a non-ST-segment elevation myocardial infarction [35]. In summary, cTNT may be helpful to detect asymptomatic CAD, especially multivessel disease, and as a predictor of mortality in CKD patients. However, as for several other markers, the variability of the test both day to day and from one laboratory to the other may limit the ability to issue widely applicable guidelines on the use this biomarker in CKD.

## 4. C-Reactive Protein

Systemic inflammation plays a major role in the development of atherosclerosis leading to coronary heart disease. C-reactive protein (CRP), an acute phase reactant, is an established marker of systemic inflammation in the general population and patients with CKD [36–38]. Elevated CRP levels, when measured using highly sensitive assays (hsCRP), are predictive of future coronary events in the general population [39]; currently a serum level of hsCRP >3 mg/L is considered a marker of high risk for events [40]. Although the clinical utility of hsCRP in CKD is still under debate, a few studies reported a significant association between hsCRP and all-cause mortality in end-stage renal failure at concentrations varying between 1 and 3 mg/L [18, 41, 42]. Apple et al. followed prospectively 399 CKD stage 5 patients in whom they measured several biomarkers. They reported that hsCRP, cTNT, and cTNI were independent predictors of death after a 2-year follow-up. NT-proBNP was only predictive when analysed as a categorical variable but not as a continuous variable [18]. Snaedal et al. measured hsCRP serially in a three-month observational study of 228 prevalent hemodialysis patients [43]. They reported that median and mean serial CRP levels were associated with an increased hazard ratio for all-cause death (1.013; 95% confidence interval, 1.004 to 1.022 and 1.012; 95% confidence interval, 1.004 to 1.020, resp.). Of interest, 13% of the patients enrolled had persistently low hsCRP levels (<5 mg/L), 19% had CRP levels > 10 mg/L, and 68% demonstrated fluctuating values. Hence, in the majority of hemodialysis patients, hsCRP is a “moving target” depending on a variety of inflammatory stimuli in the individual patient. This observation may be prognostically important. In fact, Nascimento et al. reported that the mortality rate in 180 hemodialysis patients followed for 21 months after 4 sequential measurements of CRP was significantly higher in patients with persistently elevated levels (34% death rate) than in those with low (8%) or intermittently elevated (14%) CRP levels [44]. Of note, the mortality rate was not statistically different between patients with low and intermittently elevated CRP levels. In another prospective observational study of 402 patients receiving peritoneal dialysis the investigators reported a 1.4% increase in mortality for each 1 mg/L increase in hsCRP level after 2 years of follow-up [45]. There are also conflicting reports on the value of CRP in CKD. Kanwar et al. measured troponin T and I and hsCRP in a cohort of 173 prevalent hemodialysis patients divided into 2 groups according to clinical characteristics: patient with CAD or CAD equivalent status (peripheral arterial disease and diabetes) and patients without any relevant prior cardiovascular history [46]. Prior history of CAD or CAD risk-equivalence was strongly predictive of mortality and hsCRP did not add to the risk stratification of these patients. On the contrary, both troponin T and I added incremental prognostic information. In the lower-risk patients hsCRP was predictive of mortality while troponins were not. In a study by Honda et al. where hsCRP, IL-6 serum albumin, and fetuin A were measured in 176 prevalent hemodialysis patients, hsCRP was found to be a good predictor of malnutrition but a weaker predictor of CVD and mortality than IL-6 [47]. Overall, hsCRP may be useful as an additional piece of information to assess risk in CKD patients, but its value needs to be carefully considered in the context of several limitations. There is likely a substantial difference in the significance of hsCRP elevation in predialysis and dialysis patients. In fact, exposure to dialysis membranes, even modern biocompatible membranes, and vascular access infections are associated with marked cytokine and hsCRP release; these events do not obviously take place in predialysis patients. hsCRP levels may be modulated by the concurrent use of statins. The actual risk may differ in patients with persistently elevated as opposed to intermittently elevated CRP levels. Finally the predictive value of hsCRP may be enhanced by combining its measurement with that of several other markers of risk [48].

## 5. Adiponectin 

Recent research has focused on visceral adipose tissue as a source of inflammation and adipocytokines secretion. Adiponectin (APN) is a protein secreted by adipocytes and has anti-inflammatory and antiatherogenic activities and enhances insulin-sensitivity [49, 50]. APN likely exerts its antiatherosclerotic activities by suppressing the release of proinflammatory cytokines such as TNF-*α* and IL-6 and stimulating the release of anti-inflammatory cytokines such as IL-10 [51, 52] and via enhancement of insulin sensitivity. Low APN levels have BEEN observed in patients with obesity, the metabolic syndrome, diabetes mellitus, CAD, and essential hypertension. In contrast, plasma levels of APN in patients with CKD are increased up to threefold the physiological levels [53–55], most likely due to reduced clearance or catabolism [56]. In the general population low APN levels have been associated with the development of cardiovascular disease [57, 58]. A few observational studies linked APN to adverse cardiovascular outcomes in patients with CKD [59, 60]. Becker and coworkers evaluated 227 nondiabetic patients with CKD and 76 healthy subjects of similar age, sex, and body mass index [61]. After a mean follow-up of 54 months, they concluded that, despite higher serum levels than in control subjects, relatively low plasma APN levels were predictive of cardiovascular events among patients with mild to moderate CKD. Similarly, Zoccali et al. reported that plasma APN levels were 2.5 times higher (*P* < 0.0001) among dialysis patients (15.0 ± 7.7 *μ*g/mL) than among healthy subjects (6.3 ± 2.0 *μ*g/mL), but the APN levels were lower among the dialysis patients who developed cardiovascular complications than those who remained event free [62]. The increased risk of cardiovascular events in CKD patients with lower APN concentrations (*P* < 0.05) relative to other CKD patients was unchanged after adjusting for multiple traditional and CKD-specific risk factors. Each 1 *μ*/mL increase in APN concentration was associated with a 3% reduction in risk of cardiovascular events.

In contrast, a subanalysis of the modification of diet in renal disease (MDRD) database performed in 820 patients with CKD showed a direct correlation between increased APN plasma concentration and the relative risk of cardiovascular mortality [63]. In multivariable adjusted Cox models, 1 *μ*/mL increase in APN was associated with a 3% (hazard ratio 1.03; 95% CI 1.01 to 1.05; *P* = 0.02) increased risk of all-cause and 6% (hazard ratio 1.06; 95% CI 1.03 to 1.09; *P* < 0.001) increased risk of cardiovascular mortality. A potential explanation for these apparently conflicting data is the reported association between increased APN concentrations and poor nutritional status in CKD [64]. However, the existence of a link between higher APN levels and increased cardiovascular risk in CKD remains to be clarified. In view of the bidirectional association of APN with events, its role as a useful marker of cardiovascular risk in CKD remains uncertain pending accumulation of further evidence.

## 6. Leptin

Leptin is a single-chain 16-kDa protein whose production is under the control of the obese (*ob*) gene. It is a neurotransmitter entirely produced in adipocytes, with the fundamental function of controlling appetite, regulating food intake, and energy expenditure [65]. Leptin is also a proinflammatory cytokine and is predominantly cleared from the circulation by the kidneys via a combination of glomerular filtration and tubular degradation [66]. Leptin levels increase in proportion to insulin levels, glucocorticoids, cytokines, and obesity [67]. In humans leptin is an anorectic agent and decreases appetite by decreasing the level of neuropeptide Y, a potent stimulator of food intake, and increasing alpha-melanocyte-stimulating hormone (alpha-MSH), an inhibitor of food intake, respectively [68, 69]. In healthy subjects and patients with type 2 diabetes mellitus, weight loss has been shown to be accompanied by a reduction in leptin serum concentration [70, 71]. These data suggest that high leptin concentrations are a component of the metabolic syndrome and may have a role in increasing the cardiovascular risk of these patients. Leptin levels are significantly elevated in patients with CKD, particularly in those undergoing dialysis compared with nondialysis patients [72, 73]. Besides decreased renal clearance other factors affect leptin levels in CKD. Metabolic acidosis reduces the release of leptin from adipocytes and uremic factors of unclear origin reduce leptin gene expression in adipocytes probably as a negative feedback due to decreased elimination [74, 75].

Leptin is believed to have proatherogenic effects that include the development of hypertension, oxidative stress, endothelial dysfunction, inflammation, and proliferation of vascular smooth muscle cells [64, 76]. High leptin levels induce activation of the sympathetic nervous system both via central and peripheral mechanisms. Several studies linked increased leptin and early markers of arterial disease such as increased carotid intima media thickness and decreased arterial distensibility [77–79]. Aguilera et al. described an association of serum levels of leptin with elevated lipids concentration and LVH in a small peritoneal dialysis cohort [80]. In a small size cohort of kidney transplant recipients (*n* = 74) there was a positive association between elevated serum leptin levels and increased peripheral arterial stiffness [79].

At this time there are sparse and conflicting data on the role of leptin as a marker of risk in CKD. Scholze et al. reported an association of leptin serum levels with cardiovascular events in 71 prevalent hemodialysis patients followed for 83 months [81]. The serum level of leptin was significantly lower in patients who died from cardiovascular complications (4.7 ± 9.4 microg/L, *n* = 32) than in survivors (7.7 ± 7.8 microg/L; *n* = 23; *P* = 0.003). Additionally, survival was shorter in patients with leptin concentrations below the median (<2.6 microg/L). Two other studies reported that leptin did not add incremental prognostic value for all-cause mortality and cardiovascular morbidity in 101 hemodialysis patients followed for a period of 4 years [82] and 181 patients followed for 3 years [83]. Hence, the value of leptin as a marker of risk remains unclear in CKD.

## 7. Fibroblast Growth Factor 23 and Klotho

It has long been recognized that secondary hyperparathyroidism (SHPT) is a consequence and a complication of CKD. The global syndrome that involves bone and vascular disease has been aptly termed CKD-mineral bone disorder (CKD-MBD) [84]. SHPT is a complex process that involves numerous steps such as vitamin D deficiency, hypo- and/or hypercalcemia, and hyperphosphatemia. Phosphorus levels were believed to be under the direct control of the parathyroid glands until the fairly recent discovery of a new hormone called fibroblast growth factor 23 (FGF-23). FGF-23 is a 32-kD protein secreted by bone osteocytes; its primary function is to promote phosphaturia by suppressing the expression of sodium-phosphate cotransporters NaPi-2a and NaPi-2c in the proximal tubule [85, 86]. In addition, FGF-23 acts as a counter-regulatory hormone for vitamin D by blocking the generation of 1,25(OH)2D both through inhibition of the renal 1*α*-hydroxylase enzyme and through the stimulation of the 24-hydroxylase enzyme that is responsible for the degradation of both the 25(OH)D and 1,25(OH)2D metabolites [87]. Klotho acts as a coreceptor for FGF-23 and its presence appears to be mandatory to induce FGF-23-specific signaling pathways in the kidney, parathyroid glands, and other tissues [88, 89]. Once Klotho is shed from the cell by proteolytic cleavage, it is released in the circulation and serves as a phosphaturic and hypocalciuric hormone independent of FGF-23 [90].

However, it has been postulated that FGF-23 may also operate via Klotho independent mechanisms. In the acute kidney injury (AKI) setting, FGF-23 elevation may precede creatinine elevation and phosphate metabolism impairment [91]. Whether this is due to inflammation or a decrease in Klotho in response to acute uremia needs further elucidation.

Like phosphorus, PTH, and vitamin D, FGF-23 has been independently associated with risk of all-cause death in dialysis and CKD patients, heart failure, cardiovascular events, and death in the general population [92, 93]. A cross-sectional study in 177 patients with mild to moderate CKD submitted to coronary angiography found that FGF-23 increases early in the course of CKD and, as shown for other bone metabolism hormones (PTH, Fetuin A), it is an independent predictor of CAD severity after adjusting for traditional risk factor [94]. Seiler et al. measured plasma FGF-23 levels in 149 CKD patients not receiving dialysis [95]. Patients were stratified according to the median baseline FGF-23 levels (>104 versus ≤104 rU/mL) and were followed for an average of 4.8 ± 0.9 years till the first occurrence of a cardiovascular event. At baseline, patients with more advanced CKD demonstrated higher FGF-23 serum levels. Traditional cardiovascular risk factors and prevalent cardiovascular disease did not differ between groups. Fifty patients experienced a cardiovascular event during follow-up. Compared to CKD patients with an FGF-23 level below the median, those with levels above the median experienced a higher rate of events [HR 2.49 (95% CI 1.40–4.39); *P* = 0.002]. In the Chronic Renal Insufficiency Cohort (CRIC), a graded and independent association between high levels of FGF-23 and CKD progression, congestive heart failure, and atherosclerotic events (myocardial infarction, stroke, and peripheral vascular disease) was documented among 3860 CKD 2–4 patients [96, 97]. Serum levels of FGF-23 were measured in 13,448 subjects with preserved renal function (mean eGFR was 97 mL/min per 1.73 m^2^) enrolled in the atherosclerosis risk in communities study (ARIC) [98]. The investigators documented that among patients with an FGF-23 serum level in the top quintile (>54.6 pg/mL) compared to those in the versus first quintile (<32.0 pg/mL) the risk of ESRD increased 2-fold (HR 2.1, 95% CI: 1.31–3.36, *P* < 0.001) independent of numerous confounders. In the Heart and Soul study the investigators measured FGF-23 in 833 outpatients with stable CAD and no CKD [99]. During a follow-up of 6 years, 220 patients died and 182 suffered a CVD event. After adjusting for traditional risk factors, patients in the highest tertile of FGF-23 concentrations demonstrated a 2-fold greater risk of death (HR, 2.15 [95% CI, 1.43 to 3.24]) and CVD events (HR, 1.83 [CI, 1.15 to 2.91]).

The coreceptor Klotho has been shown to have direct vascular effects by increasing both production of reactive oxygen species in human vascular smooth muscle cells and NO production in endothelial cells. Klotho deficiency has been associated with oxidative stress and inflammation in ESRD patients [100].

In a large observational study of 804 community-dwelling elderly (age greater than 65 years), plasma Klotho was inversely and independently associated with the risk of all-cause mortality (data adjusted for age, sex, education, body mass index, physical activity, total cholesterol, high-density lipoprotein cholesterol, cognition, 25-hydroxyvitamin D, parathyroid hormone, serum calcium, mean arterial pressure, and chronic diseases). Participants within the lowest tertile of serum Klotho (<575 pg/mL) had a 78% greater risk of death than patients within the highest tertile (>763 pg/mL; hazard ratio: 1.78; 95%; CI 1.20–2.63) [101]. In a cross-sectional study of 956 individuals, Klotho gene polymorphisms were associated with occult coronary artery disease (CAD) (defined as the occurrence of a reversible perfusion defect on nuclear myocardial stress testing) independent of known risk factors for CAD [102]. Recently a significant correlation was reported between severity of CAD and reduced levels of Klotho gene expression in the aorta as well as lower serum levels of soluble Klotho [103]. In a subanalysis of the Heart and Soul study the serum levels of FGF-23 and its coreceptor Klotho lacked an association with left ventricular mass and ejection fraction in the absence of kidney disease [104].

There is no reliable assay to measure Klotho to this date and despite the above findings the evidence to support the utility of FGF-23 and Klotho as markers of risk is still inconclusive; therefore they cannot be recommended for routine clinical use.

## 8. Fetuin-A and Calciprotein Particles

The alpha 2-Heremans Schmid glycoprotein, also known as Fetuin A, is secreted by hepatocytes in greater concentration during fetal than adult life. It is believed to modulate bone formation and brain development although its full function is still unknown. Fetuin A forms soluble complexes in the circulation with calcium-phosphate crystals. The calcium-phosphate and Fetuin-A complexes form stable particles (less than 100–200 nm in diameter) called calciprotein particles (CPPs) that exist as colloids and do not precipitate [105]. A growing body of evidence suggests that CPPs may be isolated from the serum of patients with CKD but not in healthy individuals; they are further believed to mediate in part the harmful effects associated with chronically elevated phosphate in CKD [106–108]. Unlike inorganic phosphate that stimulates the expression of sodium-phosphate cotransporters across the cellular membrane, CPPs seem to induce cellular responses through an interaction with the plasma membrane and the induction of intracellular messengers but the mechanisms remain to be elucidated. In a cohort of 200 patients suffering from CKD stages 3 and 4, higher levels of CPPs were associated with hyperphosphatemia, increased levels of C-reactive protein, oxidized low density protein, bone morphogenic proteins 2 and 7 as well as a rapid decline in renal function, and a greater aortic pulse wave velocity ((PWV) beta coefficient 0.059, *P* = 0.016, *R* ^2^ = 0.362) which is known to be a strong predictor of major CV events in CKD patients [107].

The use of CPPs has also been proposed as a simple predictor of the overall uremic milieu propensity for calcification [106]. The clinical validity of such test has been assessed in a recent series of 184 CKD stages 3 and 4 patients. Descending tertiles of T_50_ (a measure of the calcifying propensity of a patient's serum) were associated with higher aortic PWV as well as all-cause mortality, independent of several traditional and CKD specific risk factors [109].

Although intriguing, more studies are necessary to further elucidate the role of CPPs as a key mediator of CV damage and as a potential therapeutic target in CKD patients.

## 9. Wingless (Wnt) Antagonists Inhibitors

Wingless (Wnt) antagonist inhibitors such as sclerostin and Dickkopf-1 (DKK-1) are newly described factors involved in the bone-vascular axis [110]. Wnt activation promotes osteoblast and suppresses osteoclast activity by increasing the ratio of osteoprotegerin (OPG) to receptor activator of nuclear factor-kappaB ligand (RANKL). This leads to bone mineralization and bone turnover downregulation. The Wnt pathway is also involved in vascular and cardiac valve calcification and preliminary data suggest that its overexpression may be identified within ectopic calcification or during calciphylaxis [111, 112]. Reduced Wnt signaling seems to occur in the earliest phases of CKD likely due to the increased expression of Sclerostin and DKK-1 and it may be responsible for accelerated loss of bone mass and strength, uremic resistance to PTH, and increased vascular calcification [110, 113, 114].

Nonetheless, the clinical significance of sclerostin as a marker of risk remains unclear. In a series of 140 patients with CKD stages 2–5D [115], advancing stages of CKD were accompanied by a graded increase in serum levels of sclerostin. At this stage it is unclear whether this is due to reduced renal clearance or increased production in CKD. In the study by Desjardins et al. [115], sclerostin levels were associated with serum levels of phosphate, FGF-23, and IL-6 as well as protein bound uremic solutes such as indoxylsulphate and p-cresyl sulphate and were independent predictors of arterial stiffness and mortality, although they were not associated with vascular calcification. Further adjustments for confounders significantly attenuated the strength of the association of sclerostin with mortality [115].

In a post hoc analysis of 100 prevalent hemodialysis patients, higher levels of serum sclerostin were associated with decreased mortality (age and sex adjusted HR: 0.33, 95% CI 0.15–0.73; *P* = 0.006) [116]. After further adjustment for bone specific alkaline phosphatase, serum sclerostin levels dropped out of the multivariable model as a significant variable.

Drechsler and coworkers documented an independent inverse association of sclerostin with cardiovascular (HR 0.29; 95% CI 0.13–0.62) and all-cause mortality (HR 0.39; 95% CI: 0.22–0.68) in 673 hemodialysis patients enrolled in the NECOSAD study [117]. The association tended to lose statistical significance when the observation was prolonged past the first 18 months of dialysis. At the current stage of knowledge, therefore, the role of Wnt signaling inhibitors as markers of risk needs further elucidation and confirmation.

## 10. Neutrophil Gelatinase-Associated Lipocalin

Neutrophil gelatinase-associated lipocalin (NGAL) is a 25–135 kDa (depending on the tertiary structure of the peptide) member of the lipocalin iron-carrying proteins family [118] and is highly expressed in kidney following ischemic or nephrotoxic injury [119–121]. Although NGAL was originally isolated in neutrophils [122], numerous other tissues express this protein, namely, kidney, liver, and vascular cells (endothelial, smooth muscle cells, and macrophages in atherosclerotic plaques), as well as cardiomyocytes [123–125].

NGAL inhibits the degradation of gelatinase B leading to enhanced proteolytic activity with collagen degradation effects [123]. At steady state, NGAL concentration is negligible (about 20 ng/mL) in both blood and urine [126]. These concentrations likely reflect neutrophils production and renal clearance, the major regulators of NGAL concentration at steady state. As renal function declines, NGAL levels increase and they become markedly increased in chronic dialysis. However, high-flux hemodialysis may significantly remove NGAL from plasma [127].

Identification of increased levels of NGAL in blood and urine in several renal disease states has generated an interest in NGAL as an early marker of acute kidney injury [128]. A recent meta-analysis of 19 studies including 2538 patients from 8 countries summarized the available evidence regarding NGAL as a biomarker [129]. Although data were mainly derived from patients undergoing cardiac surgery, plasma NGAL was a strong predictor of acute kidney injury, initiation of renal replacement therapy, and in-hospital mortality with odds ratios ranging from 8.8 to 18.6 for high versus low NGAL [129].

Although NGAL was associated with progression of CKD after kidney injury in a few reports [130–133], an analysis from the Chronic Renal Insufficiency Cohort (CRIC) questioned its role as a useful biomarker to predict progression [134]. In the CRIC cohort of 3386 patients suffering from CKD 2–4, NGAL was an independent predictor of worsening of CKD (defined as a 50% eGFR decrease or dialysis initiation) but it did not add significantly to the discrimination model suggesting a marginal value of urinary NGAL as a predictor of CKD progression [134].

Investigators from Japan recently reported on the use of NGLA as a marker of CV risk in 88 hemodialysis patients [135]. At the end of 1-year follow-up they recorded 20 events; patients with event had significantly higher levels of NGAL and pro-BNP at study inception than event-free survivors (357 ± 21 versus 290 ± 11 ng/mL and 553 ± 119 versus 172 ± 73 pg/mL, *P* < 0.01 for NGAL and proBNP, resp.). After adjustment for confounders each 1 ng/mL increase in NGAL was associated with a 3% (OR: 1.03; 95% CI: 1.01–1.06) increase in the risk of CV events. Patients with simultaneous increase in NGAL and pro-BNP exhibited a 25-fold higher risk of CVD than patients with low NGAL and pro-BNP.

NGAL has been investigated as a prognostic marker in patients suffering from acute heart failure (AHF). Maisel and coworkers evaluated 186 patients admitted via the emergency department with AHF and measured NGAL and BNP [136]. At 30 days, heart failure readmission and death occurred in 29 patients; the NGAL serum level was higher in patients with events (134 versus 84 ng/mL, *P* < 0.001) and NGAL was an independent predictor of events in multivariable models (*P* = 0.001), while BNP was only of borderline significance (*P* = 0.052). The addition of NGAL to a multivariable-adjusted model lead to a 29.8% net reclassification improvement for prediction of events.

In the Rancho Bernardo Study, NGAL was associated with inflammation, lower HDL-cholesterol levels, and lower creatinine clearance [137]. In this study each standard deviation increase in log-transformed NGAL level was associated with a significant increase in all-cause mortality (HR 1.19; 95% CI: 1.07–1.32), CV death (HR 1.33; 95% CI: 1.12–1.57), and the combined endpoint (HR 1.26; 95% CI: 1.10–1.45).

In summary, the limited available evidence suggests that NGAL is a weak marker of risk for renal function decline but might be a predictor of cardiac event in the setting of HF in patients with and without renal disease. As for other biomarkers, future work will need to define reliable cutoff values in different populations and standardize results arising from different available assays.

## 11. Plasma Growth Differentiation Factor-15

Plasma growth differentiation factor-15 (GDF-15) is generated as a 40-KDa propeptide from which the NH2-terminus is cleaved and the resulting 30-KDa protein is secreted as active form [138]. GDF-15 has been described as a potential inhibitor of left ventricular hypertrophy [139]. An increase in pre- or afterload in experimental settings induces an increase in plasma GDF-15 possibly through proinflammatory cytokines and oxidative stress-dependent signaling pathways [140, 141]. Several studies have shown that GDF-15 is over expressed in patients suffering from atherosclerotic diseases such as myocardial infarction, stroke, and others types of atherosclerotic disease [142–144]. The literature regarding GDF-15 in patients affected by CKD is limited. Interesting data were derived from the 2,614 participants in the Framingham Offspring study followed for a mean of 9.5 years [145]. The investigators reported that higher plasma levels of GDF-15 were associated with incident CKD (multivariable-adjusted OR 1.9 per 1-unit increase in log-GDF-15, 95% CI 1.6–2.3, *P* < 0.0001) and a rapid decline in kidney function (OR 1.6, 95% CI 1.3–1.8, *P* < 0.0001). In a prospective observational study Lajer et al. showed that GDF-15 was an independent predictor of all-cause and cardiovascular mortality (HR 3.6 [95% CI 1.3–10.3]; *P* = 0.014) and worsening renal function in 451 patients with diabetic nephropathy [146]. Additionally, a study originally designed to evaluate GDF-15 for prognostication of cardiovascular and cancer morbidity and mortality in 940 older men reported a close association between the highest GDF-15 tertile and decline in renal function [147]. Although this marker is of some interest there is a need for substantially more research in patients with renal failure.

## 12. Asymmetric Dimethylarginine

As an endogenous inhibitor of nitric oxide (NO) synthases, asymmetric dimethylarginine (ADMA) causes endothelial dysfunction, vasoconstriction, elevation of blood pressure, and aggravation of experimental atherosclerosis [148, 149]. After its release from methylated proteins, circulating ADMA levels are regulated via glomerular filtration and enzymatic degradation by dimethylarginine dimethylaminohydrolase. Originally described by Vallance and colleagues [150], ADMA has been investigated fairly extensively in the CKD population, especially in dialysis. Although the exact mechanism linking ADMA with CVD risk in dialysis patients remains to be clarified, one of the potential mechanisms involves an interaction with the sympathetic nervous system [151, 152]. Inhibition of NO synthesis by ADMA leads to enhanced norepinephrine release from sympathetic nerve endings, whereas sympathetic activation impairs endothelium-dependent NO dilation [153, 154]. Zoccali et al. reported that ADMA is an independent predictor of cardiovascular events (HR: 1.17, 1.04–1.33, *P* = 0.008) and mortality (HR 1.26, 95% Cl 1.11–1.41, *P* = 0.0001) in patients receiving chronic hemodialysis [155]. The same researches showed that ADMA is associated with progression of carotid intima media thickness, cardiac remodeling, and LVH in hemodialysis [156, 157]. In patients with mild to moderate CKD, patients with levels of ADMA above the median (0.46 ± 0.12) showed a faster decline in renal function (*P* < 0.0001), and their mean time to end-stage renal failure was significantly shorter than in patients with ADMA levels below the median (52.8 months (95% CI 46.9 to 58.8) versus 71.6 months (95% CI 66.2 to 76.9)) [158]. The risk of doubling the serum creatinine level or the need for renal replacement therapy increased by 47% for every 0.1 *μ*mol/L increase in ADMA concentration. In contrast, Busch et al. did not find any predictive role for ADMA in a heterogeneous group of patients suffering from CKD [159].

## 13. Paraoxonase 1

Paraoxonase 1 (PON1) is a 354-amino acid HDL-associated enzyme and has been shown to reduce the susceptibility of LDL to peroxidation [160]. This activity may provide antiatherogenic protection.

Juretić et al. reported a significantly lower concentration of PON1 in 69 patients receiving chronic hemodialysis compared to 145 controls [161]. Similarly, Dirican and coworkers found PON1 levels to be significantly lower in predialysis (*n* = 28) and hemodialysis (*n* = 44) patients compared to controls (*n* = 26) [162]. Serum PON1 activity correlated inversely with serum urea and Cr levels. Subsequently, Saeed et al. showed that PON1 serum level was the best predictor of carotid IMT in a small cohort of predialysis and hemodialysis patients [163]. Sztanek et al. showed that the serum PON1 concentration was lower in patients who had undergone renal transplant (*n* = 78) and those receiving chronic hemodialysis (*n* = 108) than in controls (*n* = 63, *P* = 0.05) [164]. They further described a significant correlation with other biomarkers associated with cardiovascular risk such as homocysteine (*P* = 0.003) and ADMA (*P* ≤ 0.05). Recent evidence suggests that optimization of renal replacement therapy may produce an increase in PON1 activity, due in part to removal of PON1 inhibitors by high efficiency dialysis [165].

## 14. Conclusions

It is very unlikely that any marker will have sufficient reproducibility, predictive power, and ease of accessibility to be embraced as the single best marker for prediction of untoward events in CKD. More likely and logically, a combination of biomarkers may provide useful prognostic information. Markers may also become useful as a guide for therapy to select the patient most likely to respond to an intervention with a more individualized approach. However, several methodological issues must be addressed before the routine use of serum biomarkers can be implemented. The close correlation between different biomarkers likely reflects the complexity of the pathophysiological processes leading to CVD in CKD. Although an integrated approach seems reasonable, large* ad hoc* observational studies should define the most parsimonious approach to risk prediction to avoid unnecessary and expensive testing in different clinical settings. In this regard, the lack of standardization for different assays and the use of cohort specific cutoff values to define high versus low risk subjects do not allow for a reliable comparison of different studies. Validation of cutoff values (external validation) as well as definition of the inter- and intraassay variability for each commercially available assay is a priority to understand how to use the information derived from different biomarkers. Most of all, however, biomarkers must be easy to use and understand to become tools the practicing physician can implement daily. Although promising, available data (Table 1) on serum biomarkers for risk prediction are currently insufficient to recommend their routine use for prognostication and as a guide to therapy in CKD patients.

## Figures and Tables

**Table 1:** Summary of evidence on the use of serum biomarkers for CV risk prediction in CKD.

Biomarker	Metabolism			Prediction		Clinically useful for	
	Production	Accumulation in CKD	Dialysis	Putative mechanism(s) involved	Outcome	Risk stratification	Guide therapy
Natriuretic peptides							
Atrial natriuretic peptide (ANP)	Atrial myocardium	Yes	Dialyzable only to a small degree	Marker of left ventricular dysfunction, severity of CHF in both general and CKD populations	All-cause mortality in CKD and dialysis patients. Sudden cardiac death and MI in dialysis patients	Useful	Useful (for CHF)
B-type natriuretic peptide (BNP)	Ventricular myocardium						
C-type natriuretic peptide (CNP)	Endothelial cells/myocardium						

Cardiac troponins							
Troponins T, I, and C	Striated muscle	Yes	Increased in dialysis patients	Released after cardiac injury (cTNT)	Predicts CAD and death in CKD and dialysis patients	Useful	No data
Cardiac troponin I (cTNI)	Cardiomyocytes						
Cardiac troponin T (cTNT)	Cardiomyocytes						

C-reactive protein							
C-reactive protein (CRP)	Liver-acute phase reactant	?	Increases during dialysis	Marker of systemic inflammation	Predicts CAD, all-cause mortality	Not useful if used alone	Not useful

Adiponectin							
Adiponectin (APN)	Adipocytes	Yes	? Influenced by nutritional status	Anti-inflammatory and antiatherogenic activities, enhances insulin-sensitivity. Associated with malnutrition	Cardiovascular events, all-cause mortality	Not useful, influenced by nutritional status	Not useful, influenced by nutritional status

Leptin							
Leptin	Adipocytes	Yes, glomerular filtration and tubular degradation	Increased levels influenced by insulin resistance, metabolic acidosis, and uremic toxins	Anorectic agent associated with cIMT and arterial stiffness	Inverse association with cardiovascular events?	Not useful	Not useful

Fibroblast growth factor 23 (FGF-23)							
Fibroblast growth factor 23	Osteocytes	Yes	Increased levels, though low molecular weight and dialysable	Suppresses renal tubular phosphate reabsorption, 1,25OH vitamin D activation and increases 1,25HO vitamin D degradation and possibly iPTH secretion	Associated with ESRD occurrence, CKD progression, LVH, CHF, and atherosclerotic events, all-cause mortality	Useful	No data

Klotho							
Klotho	Produced in different tissues: endothelial cells, parathyroid glands, renal tubular cells, and VSMC	Not known	Not known	FGF-23 coreceptor (see FGF-23 actions); ROS and NO production	Oxidative stress, vascular calcification, and all-cause mortality	No data	No data

Fetuin A and Calciprotein Particles							
Fetuin-A/Calciprotein Particles	Hepatocytes/serum	Yes	?	Fetuin A forms soluble complexes in the circulation with calcium and phosphate crystals to avoid their precipitation.	Mortality. CKD progression	May be useful	No data; may become useful

Wnt inhibitors							
Sclerostin	Osteocytes	Yes	Increased in dialysis patients	Inhibitors of Wnt pathway. Increase bone mass and reduce bone turnover	Mortality? CKD-MBD?	No data	No data
Dickkopf-1 (DKK-1)	?	?	?				

Neutrophil gelatinase-associated lipocalin (NGAL)							
NGAL	Several tissues	Inversely associated with creatinine clearance	?	Suppresses gelatinase B inactivation leading to proteolytic activity and collagen degradation	Acute Kidney Injury, CKD progression, CV events, and mortality	Useful	No data

Plasma growth factor-15 (GDF-15)							
GDF-15	?	?	?	Potential inhibitor of left ventricular hypertrophy, overexpressed in atherosclerotic disease	Incident CKD, all-cause and CV mortality	No data	No data

Asymmetric dimethylarginine (ADMA)							
ADMA	?	Renal clearance	Increased in dialysis patients	Impairs endothelium dependent NO dilatation	cIMT, LVH, CV events, renal function decline, and mortality	Useful	No data

Paraoxonase 1 (PON1)							
PON1	?	Inversely associated with creatinine clearance	Reduced in dialysis patients. Optimal RRT may produce an increase in PON1	HDL-associated enzyme and has been shown to reduce the susceptibility of LDL to peroxidation	cIMT, CV events	No data	No data

CHF: congestive heart failure; CAD: coronary artery Disease; cIMT: carotid intima media thickness; iPTH: intact parathyroid hormone; LVH: left ventricular hypertrophy; VSMC: vascular smooth cells; CKD-MBD: chronic kidney disease mineral bone disorder; Wnt: wingless pathway.

## Abbreviations

95% CI: 95% confidence interval
ADMA: Asymmetric dimethylarginine
ANP: Atrial Natriuretic Peptide
BNP: B-type natriuretic peptide
CAD: Coronary artery disease
CHF: Congestive heart failure
cIMT: Carotid intima media thickness
CKD: Chronic kidney disease
CKD-MBD: Chronic kidney disease mineral bone disorder
CPP: Calciprotein particles
CRP: C-reactive protein
cTNI: Cardiac troponin
CV: Cardiovascular
CVD: Cardiovascular disease
DKK-1: Dickkopf-1
FGF-23: Fibroblast growth factor 23
GDF-15: Plasma growth factor-15
HR: Hazard ratio
iPTH: Intact parathyroid hormone
LVH: Left ventricular hypertrophy
NGAL: Neutrophil gelatinase-associated lipocalin
OR: Odds ratio
PON1: Paraoxonase 1
ROC: Receiver operating characteristic
VSMC: Vascular smooth cells
Wnt: Wingless pathway.
